# The paradox of creativity in generative AI: high performance, human-like bias, and limited differential evaluation

**DOI:** 10.3389/fpsyg.2025.1628486

**Published:** 2025-08-07

**Authors:** Joy Desdevises

**Affiliations:** OCTO Technology, Accenture, Paris, France

**Keywords:** creativity, generative AI, ChatGPT, fixation effect, divergent thinking, problem-solving

## Abstract

Creativity plays a crucial role in helping individuals and organisations generate innovative solutions to arising challenges. To support this creative process, generative Artificial Intelligence (AI), such as ChatGPT is being used increasingly. However, whether such a generative AI model can truly enhance creativity or whether it exhibits similar creative biases to humans is unclear. This study, conducted in 2025, consisted of an experiment which involved ChatGPT-4o performing the egg task, a creativity task which measures fixation bias and original idea generation (expansion). The AI model's results were compared both to a sample of 47 human participants and to aggregated data from eight previous studies using the same procedure with the egg task. This dual comparison provides a comprehensive perspective on creative biases in both AI and humans at multiple levels. While ChatGPT demonstrated greater productivity than humans, it exhibited a comparable fixation bias, with most ideas falling within conventional categories. Furthermore, the model showed a limited capability to differentially evaluate originality, as it struggled to distinguish between original and conventional ideas, unlike humans who are typically able to make this distinction. In conclusion, although generative AI demonstrates impressive fluency by producing a large number of creative ideas, its inability to critically assess their originality and overcome the fixation bias highlights the necessity of human involvement, particularly for properly evaluating and filtering the ideas generated.

## 1 Introduction

Creativity is widely recognised as a key factor in innovation, enabling individuals and organisations to generate novel responses to complex and evolving challenges. It plays a crucial role in helping organisations adapt to change, solve problems, and maintain a competitive edge (Amabile, 1996, 1997; Anderson et al., 2004, 2014; Mumford and Gustafson, 1988; Sternberg and Lubart, 1995; Zhou and Lee, 2024). To stimulate creativity, organisations are increasingly exploring new tools, particularly generative artificial intelligence (AI), which has become progressively integrated into professional practises (Boston Consulting Group, 2021; Deloitte, 2023). Among these tools, ChatGPT has emerged as a major player, with over 300 million weekly users by the end of 2024 (Roth, 2024) and growing use across sectors (Lepti Digital, 2025), notably as a means to support idea generation (IBM, 2023, 2024). However, this widespread use raises a critical question: **does a generative AI like ChatGPT truly enhance creativity, or does it replicate the cognitive biases that constrain innovation?** Through an experimental study, I aim to determine the extent to which this tool can foster creativity or whether it exerts a biassed influence on our generation of ideas.

### 1.1 Concept, cognitive models, and the assessment of creativity

Creativity is defined as the ability to generate ideas or solutions that are both novel and appropriate (Amabile, 1996; Anderson et al., 2014; Barron, 1988; Lubart, 2001; Lubart et al., 2003; Mackinnon, 1962; Ochse, 1990; Runco and Jaeger, 2012; Sternberg, 1988, 1999; Sternberg and Lubart, 1991, 1995; Weisberg, 2015). It plays a fundamental role in various domains of human cognition, including problem solving, artistic production, and scientific innovation (Sawyer and Henriksen, 2024). Research in cognitive psychology has identified several processes involved in creative thinking, such as divergent thinking, which represents the ability to generate multiple potential solutions to a given problem (Guilford, 1967, 1977), cognitive flexibility (Guilford, 1967), and the capacity to redefine a problem (Ward et al., 1999, in Sternberg, Chapter 10). Finke et al. (1992) proposed one of the first cognitive models of creativity, highlighting the frequent obstacles encountered during the generation of ideas (see also Ward et al., 2004; Smith et al., 1995, 1993). According to these authors, individuals tend to follow the “path of least resistance,” which means to produce ideas or solutions grounded in the most accessible and conventional knowledge. For instance, in the “Alien task” (Smith et al., 1993; Ward, 1994), participants were asked to imagine a creature from another planet. Despite the open-ended nature of the task, most participants ended up designing creatures with Earth-like features (e.g., bilateral symmetry, eyes, limbs), often relying on their most familiar and accessible conceptual knowledge. These biassed responses are a manifestation of what is known as the “fixation effect” (Adamson, 1952; Duncker and Lees, 1945; German and Defeyter, 2000). Finke et al. (1992) and Smith et al. (1995) argue that in order to generate something genuinely new and original, it is necessary to expand the conceptual structures (i.e., mental frameworks and familiar associations that shape how we interpret and generate ideas; see Ward, 1994) and pre-existing knowledge on which these path-of-least-resistance ideas are based. This process is referred to by the authors as “conceptual expansion.” While this model has had a significant influence on subsequent research and continues to serve as a theoretical reference, it appears limited in explaining how ***individuals*** can overcome this cognitive bias. How can we bypass the fixation effect when we are required to generate ideas creatively? How can we escape the path of least resistance?

To address these questions, Cassotti et al. (2016) proposed a cognitive model of creativity inspired by dual-process theories of thinking (Houdé, 2001; Kahneman, 2011). According to this triadic model (Cassotti et al., 2016), creativity relies on the interaction of three cognitive systems:

- The intuitive system (system 1), which is fast and spontaneous, generating ideas often shaped by preexisting schemas;
- The deliberative system (system 2), which is analytical and deliberate, allowing for more in-depth and original exploration of ideas;
- The inhibitory control system, which plays a key role in suppressing ideas from the intuitive system in order to activate the deliberative system.

Cognitive inhibition thus appears to be a crucial lever for overcoming the fixation effect and promoting the emergence of creative ideas (see also Agogué et al., 2015; Beaty et al., 2014; Benedek et al., 2014; Camarda et al., 2018; Vartanian, 2009). While the triadic model highlights the importance of inhibitory control in the generation of creative ideas, it remains necessary to observe and quantify these underlying cognitive processes. Several tasks have been developed in cognitive psychology to assess the individuals' ability to overcome mental fixation (e.g., Torrance, 1966; Ward, 1994), both in convergent (i.e., focusing on finding the single best solution) and divergent thinking (i.e., generating multiple, varied ideas). However, few of these tasks allow for both an objective and systematic evaluation of the fixation effect and the originality of the ideas generated, which represent two essential dimensions of creativity. To address this gap, Agogué et al. (2014) developed a divergent thinking task based on the Concept-Knowledge (C-K) theory of Hatchuel and Weil (2003, 2009), which distinguishes between the space of existing knowledge (K) and the space of concepts (C) where novel ideas emerge. In the “egg task,” derived from this framework, participants are asked to generate as many original ideas as possible to solve the following problem: “Ensure that a hen's egg dropped from a height of 10 m does not break.” A predefined mapping based on Concept-Knowledge Theory organises possible responses into ten conceptual categories: act before the fall, act after the fall, cushion the fall, slow down the fall, use living beings, use the natural properties of the egg, modify the properties of the environment, modify the properties of the egg, interrupt the fall, and protect the egg (Agogué et al., 2014). Experimental findings show that nearly 80% of the ideas cluster around three dominant categories; cushioning the fall, protecting the egg, and slowing the fall; while the remaining seven categories are rarely explored (e.g., Agogué et al., 2014; Camarda et al., 2018, 2024; Cassotti et al., 2016; Desdevises, 2021; Desdevises and Cassotti, 2024). These three dominant categories are thus considered to reflect the fixation effect, as they contain biassed ideas grounded in the most common and easily accessible knowledge. In contrast, ideas belonging to the remaining seven categories are considered creative: they are referred to as expansion ideas, as they involve expanding commonly shared conceptual structures and pre-existing knowledge. This task therefore enables the quantification of several creativity indicators: fluency (i.e., the total number of ideas), flexibility (number of categories explored), fixation score (i.e., the number of ideas in the fixation path, within the three dominant categories), and expansivity score (i.e., the number of expansion ideas, i.e., outside fixation, within the seven categories representing expansion). Together, these indicators provide a systematic framework for assessing creative performance. By capturing complementary dimensions of idea generation, they are particularly well suited to addressing our research question. Accordingly, the egg-task was used in our experimental protocol. Beyond these core indicators, the current experimental protocol (see Desdevises and Cassotti, 2024) will also include an assessment of the confidence the model assigns to its outputs, in order to examine the potential role of metacognitive mechanisms in creative evaluation. Indeed, research on reasoning tasks involving single-solution problems has shown that individuals tend to express significantly lower confidence when providing biassed or incorrect responses compared to correct ones, suggesting that they possess metacognitive abilities enabling them to detect the presence of a conflict (De Neys, 2012). Recent studies have shown that this sensitivity to conflict also extends to creative thinking, where it helps individuals distinguish between ideas shaped by cognitive fixation and those that emerge through conceptual expansion (Camarda et al., 2024; Desdevises, 2021).

Building on these findings, and in light of the growing integration of generative AI into creative processes, a central question emerges: to what extent can a generative AI tool such as ChatGPT produce genuinely creative ideas? Does it exhibit a fixation bias similar to the bias observed in human idea generation? Can it, in turn, identify conflicts between the task instructions and its generated outputs, analogous to how humans detect cognitive conflict during idea generation? Cognitive models of human creativity, such as Cassotti et al. (2016) triadic model, emphasise the critical role of inhibitory control and metacognitive monitoring in enabling the generation of novel ideas. Given that generative AIs like ChatGPT operate as rapid-response systems with access to vast stores of information, it is entirely plausible that they may not only replicate human cognitive processes involved in creative thinking but also bypass them altogether. This dual possibility of both replicating and bypassing raises compelling and timely questions about how such systems align with, or diverge from, the cognitive underpinnings of human creativity. In this context, the growing presence of generative AI in creative domains prompts a closer examination of how these tools function, and whether they can truly replicate or surpass human creative processes.

### 1.2 Generative AI and creativity

Tools like ChatGPT are built on deep neural network architectures, which are artificial systems inspired by the human brain's processing of information (Vaswani et al., 2017). ChatGPT is powered by models such as GPT-4o, an artificial intelligence model trained on billions of texts from the internet. It works by learning the structure of human language and predicting, word by word, what is likely to come next in a sentence (OpenAI, n.d.). Therefore, models like GPT-4o can quickly generate responses based on context and produce large amounts of text, without being hindered by human cognitive limitations such as working memory or fatigue. These models represent language using mathematical structures known as vectors, which allow them to capture complex relations between words. This enables them to navigate large “semantic spaces” (i.e., mental maps of how words and ideas are related based on how they appear together in language) associating concepts that may seem distant or unconventional. As a result, they can generate both conventional and novel ideas, depending on how the prompt activates learned patterns (OpenAI, n.d.).

Historically, artificial intelligence was primarily regarded as a powerful tool for analytical and computational tasks, due to its ability to rapidly process large volumes of data and optimise complex processes (e.g., Kakatkar et al., 2020). In contrast, creative tasks were long considered to be the exclusive domain of human intelligence (Bilgram and Laarmann, 2023). However, the recent emergence of generative artificial intelligence has profoundly challenged this distinction, reshaping our understanding of the role of machines in the process of generating creative ideas. In just a few months, these systems have shifted from being mere cognitive assistants to becoming central actors in fields as diverse as art, music, writing, and innovation (Bouschery et al., 2023; Brem et al., 2021). The use of AI for creative tasks, in particular for the generation of ideas, has thus become a priority research topic, raising new questions about both its potential and its limitations (Bilgram and Laarmann, 2023). A growing body of research is now examining the creative potential of generative AI, with findings suggesting that these tools can produce creative outputs that, depending on the context, may be less effective (e.g., Stevenson et al., 2022), comparable to (e.g., Haase and Hanel, 2023), or superior to those generated by humans (e.g., Guzik et al., 2023).

Recent studies have explored the creative capabilities of generative AI models through standardised ideation tasks. For instance, the Alternative Uses Task (AUT), originally developed by Guilford (1967), requires participants to generate as many unusual or creative uses as possible for a common object (e.g., a brick or a paperclip), and is widely used to assess divergent thinking. Using this task, Stevenson et al. (2022) observed a human advantage when compared to the GPT-3 model of ChatGPT. However, more recent research highlights not only the rapid progress of AI models but also the development of strategies to overcome some limitations identified. Indeed, Summers-Stay et al. (2023) demonstrated that a “generate-and-select” approach was necessary for GPT-3 to surpass the level of human creativity, and Haase and Hanel (2023) showed that several AI chatbots, including GPT-4o, achieve creativity levels comparable to those of humans across various evaluation criteria. In another study, Guzik et al. (2023) examined the creative capacities of GPT-4o using the Torrance Tests of Creative Thinking, a set of tasks designed to assess divergent thinking and creativity through activities such as generating unusual uses for objects, completing partial drawings, or imagining the consequences of hypothetical situations. The authors found that the model performs at or above human levels across several dimensions of creativity. In terms of originality, GPT-4o generated novel and unexpected ideas that were judged to be highly creative, particularly in imaginative tasks, outperforming the human control group. The model also exhibited high fluency, producing a large number of ideas with ease. Results for flexibility were more mixed: while GPT-4o achieved high overall flexibility scores, it performed less well than humans on specific tasks requiring diverse categorical thinking.

Although some studies show that AI can surpass human performance in creativity in certain contexts or on specific measures, a key question remains: do the outputs generated by AI show evidence of a fixation effect during the creative process? In other words, when responding to a creative prompt, do the ideas produced tend to be constrained by a fixation bias present in the training data? This raises the issue of whether the ideas generated by AI are truly original and novel, or if they remain influenced by predictable and repetitive patterns observed in its training data.

In line with this, Doshi and Hauser (2024) showed that AI can enhance the perceived quality of creative storeys, particularly among less experienced authors, though often at the cost of homogenised outputs. In other words, AI-generated content tends to be more predictable and repetitive, reflecting a form of fixation bias whereby outputs are constrained by patterns in the training data, limiting diversity and originality. In addition, Agogué et al. (2014) demonstrated that exposure to an example before a creative task significantly shapes subsequent outputs: this effect was replicated and extended by Wadinambiarachchi et al. (2024), who found that AI-generated images induce a stronger fixation effect than conventional tools like Google Images, further reducing diversity and originality in visual design tasks (see also Davis et al., 2019).

Although recent studies have shown that generative AI can amplify fixation bias in humans (Wadinambiarachchi et al., 2024) and tends to generate relatively repetitive and predictable outputs (Doshi and Hauser, 2024), they have yet to provide a definitive answer regarding the intrinsic nature of these outputs: are they spontaneously original, or do they reflect fixation-driven patterns learned from training data? My study aims to address this gap by focusing on a standardised creative task, the Egg Task, to systematically compare human and AI-generated ideas. By examining the presence of the fixation effect and analysing differences in cognitive patterns, this research seeks to determine whether AI can bypass the cognitive constraints that often limit human creativity.

### 1.3 Importance of the study and hypotheses

Despite recent advancements in the study of generative AI's creative capabilities, few studies have systematically compared the cognitive processes involved in human creativity and AI performance on ideation tasks. Current research often focuses on subjective evaluations of creativity, without thoroughly exploring whether these models replicate or overcome human cognitive biases, such as the fixation effect, which limits the exploration of truly novel ideas. This study aims to address this gap by analysing the generative patterns of AI-generated creativity (ChatGPT), particularly in a standardised creative task with a problem-solving component (the egg-task) and by comparing the nature of AI-generated and human-generated ideas. Therefore, the objective of my study is to compare the performance of GPT-4o and humans on five key dimensions of creative thinking:

1. **Fluency (the total number of ideas generated):** language models like ChatGPT are designed to produce large volumes of content without being constrained by cognitive fatigue or working memory, unlike humans. It is therefore expected that GPT-4o will generate a higher number of ideas than individuals in the egg task.
2. **Fixation score (the number of ideas in the fixation path, within the three dominant categories):** due to its operation based on predicting the most probable content (OpenAI, n.d.), ChatGPT may replicate cognitive biases similar to those observed in humans, such as the fixation effect. I therefore anticipate that, like humans, ChatGPT will predominantly generate ideas from the three dominant categories (i.e., fixation).
3. **Expansivity score (the number of expansion ideas, i.e., outside fixation, within the seven categories representing expansion):** leveraging its capacity to navigate broad semantic networks using vector-based representations (OpenAI, n.d.), ChatGPT may be able to produce more distant or atypical associations. If this ability enables it to deviate from the most statistically common associations, it should generate a greater number of expansive ideas than human participants.
4. **Category diversity (number of categories explored out of the 10 existing):** in human creativity research, this dimension is referred to as cognitive flexibility. However, since no cognitive process is involved in ChatGPT's generation mechanism, we refer here to “category diversity.” Given its ability to navigate vast semantic spaces, ChatGPT should be capable of generating both conventional and novel ideas by associating distant concepts (OpenAI, n.d.). This ability may allow it to explore more categories than human participants. However, as it operates by predicting the most likely subsequent words based on patterns learned from its training data, it may also favour more common, predictable associations. This reliance on statistical probabilities could limit its exploration of less typical or more diverse ideas. This measure of category diversity will be assessed to determine whether ChatGPT explores a broader range of categories than humans or if it is constrained by more typical associations.
5. **Conflict detection (subjective creativity ratings):** I'm interested in the AI's ability to evaluate its own outputs. GPT-4o is regarded as a strong model for reference-free evaluation, capable of providing consistent assessments of generated content when compared to weaker models (e.g., Dubois et al., 2024; Hu et al., 2024). However, it is also possible that ChatGPT (GPT-4o) may not clearly distinguish between fixation and expansion ideas, attributing similar value to both. Indeed, previous studies have pointed out that ChatGPT may exhibit evaluation biases, such as favouring longer responses over shorter ones, possibly because longer outputs are perceived as more elaborate or informative (e.g., Dubois et al., 2024; Hu et al., 2024). The creativity scores attributed by ChatGPT to its own ideas will allow us to examine a form of differential and simulated evaluation functionally analogous to metacognitive assessment in humans, shedding light on a dimension of creative processing that remains unexplored in the context of generative AI. Here, “conflict detection” is used in a strictly functional sense to describe discrepancies between generated ideas (e.g., fixation) and task instructions (e.g., the expectation of originality), without implying any underlying cognitive processes.

Thus, the current study aims to empirically test these hypotheses using a rigorous experimental approach. It will provide essential insights into how generative AI compares to human abilities in the generation of ideas and its potential to be a tool for enhancing creativity.

## 2 Method

### 2.1 Sample

The data analysed in this study were collected from two distinct sources of idea generation: human participants and ChatGPT-4o.

For the human data, secondary data were used, originally collected as part of a previous study (Desdevises and Cassotti, 2024). A sample of 47 participants from the control group of that study was selected (Age: *M* = 19.04, *SD* = 1.10; 89% women). All participants completed the creative problem-solving task commonly referred to as the egg task (Agogué et al., 2014). In addition to these individual-level data, a set of weighted average scores was computed based on aggregated results reported across 8 prior studies (5 peer-reviewed journal articles and 3 academic thesis studies; see Table 1). Comparisons to aggregated human data from prior literature are exploratory in nature and aim to provide a normative context for interpreting ChatGPT's performance, rather than supporting strict inferential claims. Only data from control groups (i.e., participants who received no experimental manipulation) were considered. All selected samples completed the task individually, using a paper-and-pencil format, with identical instructions and a strict 10-min time frame. The participants across all selected samples were novices and similar in ages, between 19 and 29 years (*M* = 21.04, *SD* = 3.96).

**Table 1:** Summary of measures reported in human control groups used as references for comparative analyses.

**Study**	**Sample size (*n*)**	**Fluency**	**Flexibility**	**Number of fixation and expansion ideas or % of fixation ideas**
Agogué et al. (2014)	28	X		X
Agogué et al. (2014)	94			X
Agogué et al. (2015)	19	X		X
Desdevises (2021) (study 1)	84	X	X	X
Desdevises (2021) (study 3)	49	X	X	X
Desdevises (2021) (study 4)	26	X	X	X
Desdevises and Cassotti (2024)	47	X	X	X
Kruse et al. (2023)	52	X	X	X

For ChatGPT, 12 distinct runs were exectued using private browser windows and different user accounts and IP addresses to minimise any potential algorithmic interference related to user identification. All outputs were produced by the same underlying model (ChatGPT-4o). While these 12 initial runs are not independent in the human sense, they capture the stochastic variability inherent to large language models (i.e., variability resulting from controlled randomness in the generation process; Chen et al., 2021). Each output was therefore treated as a distinct and plausible prompt-response interaction that a typical user could receive under identical prompt conditions. This practise aligns with current approaches in LLM evaluation, where multiple runs are used to assess the model's behavioural range (e.g., Binz and Schulz, 2023; Chen et al., 2021). To address the relatively small sample size (*n* = 12) and enable robust statistical comparisons with human data, two complementary non-parametric bootstrapping procedures were applied. To ensure transparency and reproducibility, the full Jupyter notebook with the Python scripts is publicly available on the Open Science Framework (OSF) at https://osf.io/cqpmr/?view_only=ef11630f521a4cdd95dd9200e1c7ea40. In the first approach, designed to compare ChatGPT's output to individual-level human data (i.e., 47 participants who completed the same task), a separate bootstrap procedure was performed for each variable. This involved generating 50 individual bootstrap values per variable by randomly sampling one value (with replacement) from the original 12 ChatGPT scores. This method produced synthetic individual-level distributions that allowed for direct statistical comparison with human participants using non-parametric tests. In the second approach, aimed at comparing ChatGPT's performance to aggregated human reference values (i.e., weighted means based on prior studies), 50 bootstrap observations were created by randomly resampling, with replacement, from the 12 original ChatGPT runs. For each resampled dataset, the mean value was calculated across the seven creativity-related variables, resulting in 50 aggregated bootstrap observations. This procedure enabled estimation of the central tendency and variability of ChatGPT's overall performance while preserving the characteristics of the original distribution.

### 2.2 Material and procedure

The task used in this study was a classic creative problem-solving task, the egg task, in which participants, or in this case ChatGPT, were asked to generate as many creative ideas as possible to solve the following problem: “Ensure that a hen's egg dropped from a height of 10 m does not break” (Agogué et al., 2014; Cassotti et al., 2016). An additional prompt was provided at the end of the creative task to assess ChatGPT's ability to evaluate the creativity of its own outputs. It was asked to assign a creativity score to each idea on a scale from 1 to 7 (with 1 corresponding to “not at all creative” and 7 to “highly creative”).

The prompts translated from French and submitted to ChatGPT during the experiment:

**Prompt 1:** You are a designer and are asked to propose as many original solutions as possible to the following problem: “Make sure that a hen's egg, dropped from a height of 10 m, does not break.”
**Prompt 2:** You may now assign a creativity score to each of your ideas, from 1 to 7 points (1 = not at all creative, 7 = highly creative).

## 3 Results

### 3.1 Data analysis plan

Firstly, I conducted descriptive analyses solely on ChatGPT's outputs (*n* = 12). These analyses aimed to characterise the model's performance across various creativity metrics (e.g., fluency, category diversity, fixation scores, expansion scores, and subjective creativity ratings) independently of any human comparison. This step served to establish a baseline understanding of the generative model's behaviour and output structure.

Then, I performed two complementary comparative set of analyses between ChatGPT and human data: one using individual-level data from 47 participants (Desdevises and Cassotti, 2024), and another using aggregated data from 8 prior studies. Statistical methods were chosen based on data distribution and variance assumptions.

All analyses were conducted using Jamovi software (Version 2.3.21; The Jamovi Project, 2023), with a statistical significance threshold set at α = 0.05.

### 3.2 ChatGPT-4o performance analysis

Normality tests (Shapiro-Wilk) indicated that the data corresponding to fluency, category diversity, and expansion score were not normally distributed (*p* < 0.05). Given this assumption cheque not met and the limited sample size (*n* = 12), the median was used as the measure of central tendency, and the interquartile range (IQR) was reported to describe variability (see Table 2). Furthermore, since the 12 prompt-response interactions produced by ChatGPT do not represent independent observations from a randomly sampled population, but rather multiple outputs from a single model instance, this further supports the choice of non-parametric descriptive statistics. Consequently, non-parametric tests (Wilcoxon tests) were used when necessary, and effect sizes were assessed using biserial rank correlations (*rb*).

**Table 2:** Comparison of median and interquartile ranges between bootstrapped ChatGPT scores (*n* = 50) and human participants (*n* = 47) for creativity measures.

**Measure**	**Median for ChatGPT (IQR)**	**Median for human participants (*n* = 47)**
Fluency	30.00 (25.00–30.00)^***^	7.00 (6.00–9.00)
Category diversity (/10)	6.00 (6.00–7.00)^***^	4.00 (3.00–5.00)
Number of fixations	22.50 (18.00–27.00)^***^	5.00 (4.00–6.00)
Number of expansions	6.00 (4.00–10.00)^***^	2.00 (1.00–3.00)
Subjective creativity ratings for fixations (/7)	5.22 (5.19–5.26)^***^	3.92 (3.23–4.73)
Subjective creativity ratings for expansions (/7)	5.27 (5.07–5.48) ^***^	4.21 (3.33–5.00)
Proportion of fixations (%)	80.2 (66.7–83.4) NS	71.40 (57.70–84.50)

Asterisks indicate the significance levels of Mann–Whitney U tests comparing ChatGPT-4o's bootstrapped scores to individual human participants' scores: ^***^p < 0.001. NS, not significant (p ≥ 0.05).

**Fluency**. The median number of ideas generated by the generative AI model GPT-4o is 28.5 (IQR = 24.3–30).

**Category diversity**. The median number of categories used by GPT-4o (out of 10 possible categories) is 6 (IQR = 5.75–7).

**Fixation and expansion scores**. The fixation score (number of ideas within the three dominant categories) was significantly higher (Mdn = 28.5, IQR = 18–24) than the expansion score (i.e., number of ideas within the seven original categories) (Mdn = 6, IQR = 4–8.5), with a Wilcoxon test result of *W* = 78, *p* = 0.002, *rb* = 1. This represents a median of 80.2% (IQR = 66.83.4) ideas within the fixation path.

**Subjective creativity ratings (conflict detection):** GPT-4o rated its fixation ideas (Mdn = 5.23, IQR = 5.18–5.28) as equally creative as its expansion ideas (Mdn = 5.34, IQR = 5.17–5.49), *W* = 26, *p* > 0.05. The median confidence score was 5.29 (IQR = 5.18–5.39).

### 3.3 Comparative data analyses: ChatGPT-4o compared to humans

To provide a multi-level comparison between generative AI and human performance, two complementary analytical approaches were employed. In the first approach, ChatGPT's bootstrap observations were generated by resampling each creativity variable separately (*n* = 50) and compared to individual-level data from 47 human participants (Desdevises and Cassotti, 2024). For each dependent variable, assumptions of normality (Shapiro–Wilk test) and homogeneity of variances (Levene's test) were assessed. Given that none of the variables met the assumption of normal distribution and unequal variances were observed across sources, non-parametric Mann–Whitney *U* tests were used to ensure robustness and consistency across comparisons. In the second approach, bootstrap observations were generated by resampling the entire dataset of ChatGPT responses (*n* = 50) and compared to weighted average scores derived from 8 prior studies (see Table 3). As the Shapiro–Wilk tests confirmed the normality of the resampled distributions, one-sample *t*-tests were used to compare ChatGPT's performance to these aggregated normative values.

**Table 3:** Comparison of weighted human reference values and bootstrapped ChatGPT scores (*n* = 50) for creativity measures.

**Measure**	**Reference studies (total number of human participants)**	**Mean for ChatGPT (SD)**	**Weighted mean for human participants (weighted SD)**
Fluency	7 (*n* = 305)	27.5 (1.27)^***^	7.91 (4.49)
Category diversity (/10)	5 (*n* = 258)	6.09 (0.25)^***^	5.52 (1.83)
Number of fixations	6 (*n* = 277)	21.10 (1.55)^***^	5.87 (3.85)
Number of expansions	6 (*n* = 277)	6.44 (0.71)^***^	2.32 (1.94)
Subjective creativity ratings for fixations (/7)	4 (*n* = 246)	5.23 (0.04)^***^	3.65 (1.21)
Subjective creativity ratings for expansions (/7)	4 (*n* = 246)	5.35 (0.07)^***^	4.11 (1.33)
Proportion of fixations (%)	8 (*n* = 399)	76.20 (3.04)^***^	72.06 (NA)

Asterisks indicate the significance level of one sample t- tests comparing ChatGPT-4o's bootstrapped scores to the weighted human participants' scores: ^***^p < 0.001. NS, not significant (p ≥ 0.05).

**Fluency**. ChatGPT generated significantly more ideas (Mdn = 30.00, IQR = 25.00–30.00) than the median number of ideas produced by the 47 human participants (Mdn = 7.00, IQR = 6.00–9.00), *U* = 0.00, *p* < 0.001, *r* = 1. This effect remained consistent when comparing ChatGPT's scores (*M* = 27.50, *SD* = 1.27, 95% CI [27.20, 27.90]) to the weighted human average (*M* = 7.91, *SD* = 4.49), *t*(49) = 109.00, *p* < 0.001, *d* =15.50.

**Category diversity**. ChatGPT explored a higher number of categories out of the 10 categories (Mdn = 6.00, IQR = 6.00–7.00) compared to the human participants (Mdn = 4.00, IQR = 3.00–5.00), *U* = 361.00, *p* < 0.001, *r* = 0.69. This effect is consistent when comparing ChatGPT's score (M = 6.09, SD = 0.25, 95% CI [6.02, 6.16]) to the weighted human average (*M* = 5.52, *SD* = 1.83), *t*(49) = 16.30, *p* < 0.001, *d* = 2.30.

**Fixation scores**. ChatGPT generated more ideas belonging to the fixation effect (Mdn = 22.50, IQR = 18.00–27.00) than the human participants (Mdn = 5.00, IQR = 4.00–6.00), *U* = 0.00, *p* < 0.001, *r* = 1. This result was confirmed when comparing ChatGPT's score (*M* = 21.10, *SD* = 1.55, 95% CI [20.60, 21.50]) to the weighted average of human participants (*M* = 5.87, *SD* = 3.85), *t*(49) = 69.40, *p* < 0.001, *d* = 9.82.

**Expansion scores**. ChatGPT generated more ideas belonging to the expansion (Mdn = 6.00, IQR = 4.00–10.00) than the 47 human participants (Mdn = 2.00, IQR = 1.00–3.00), *U* = 216.00, *p* < 0.001, *r* = 0.80. This effect was confirmed in the second approach, comparing ChatGPT's score (*M* = 6.44, *SD* = 0.71, 95% CI [6.23, 6.64]) to the weighted mean of human participants (*M* = 2.33, *SD* = 1.94), *t*(49) = 40.90, *p* < 0.001, *d* = 5.78.

To explore the difference between ChatGPT and humans in the proportion between fixation and expansion, I also conducted a test based on the proportion of fixation ideas relative to the total number of ideas generated. This proportion did not significantly differ between ChatGPT (Mdn = 78.60%, IQR = 66.70–81.8%) and humans (Mdn = 71.40, IQR = 57.70–84.50), *U* = 991, *p* = 0.18. This result was inconsistent when comparing ChatGPT's score (*M* = 76.20, *SD* = 3.04, 95% CI [75.3, 77.1]) to the weighted average of human participants (*M* = 72.06%), *t*(49) = 9.64, *p* < 0.001, *d* = 1.36.

**Subjective creativity ratings (conflict detection):** ChatGPT rated its own fixation ideas (Mdn = 5.22, IQR = 5.19–5.26) and expansion ideas (Mdn = 5.27, IQR = 5.07–5.48) as significantly more creative than the ratings of human participants (fixation: Mdn = 3.92, IQR = 3.23–4.73; Expansion: Mdn = 4.21, IQR = 3.33–5.00), *U* = 195.00, *p* < 0.001, *r* = 0.84 and *U* = 520.00, *p* < 001, *r* = 0.56 respectively. These differences were also confirmed when comparing ChatGPT's scores (fixation: M = 5.23, SD = 0.04, 95% CI [5.22, 5.24]; expansion: *M* = 5.35, *SD* = 0.07, 95% CI [5.33, 5.37]) to the weighted means of human participants (fixation: *M* = 3.65, *SD* = 1.21; expansion: *M* = 4.11, SD = 1.33), *t*(49) = 302.00, *p* < 0.001, *d* = 42.80; and *t*(49) = 182.00, *p* < 0.001, *d* = 25.70, respectively.

## 4 Discussion

### 4.1 Study's objective

The aim of this study was to investigate whether a widely used generative artificial intelligence system, ChatGPT, is capable to produce creative ideas in a problem-solving context, and whether its outputs exhibit the same types of cognitive biases that typically constrain human creativity and innovation. Using the Egg Task, a problem-based creativity task, I analysed key indicators of idea generation: fluency (the total number of ideas generated), fixation score (ideas falling within stereotypical or dominant categories), expansivity score (ideas falling outside of these dominant paths), cognitive flexibility (the number of distinct conceptual categories explored out of ten), and conflict detection (i.e., subjective creativity ratings).

### 4.2 Summary of the results

The findings across both bootstrapping approaches converge on nearly all key creativity metrics: fluency, category diversity, originality, the absolute number of fixation and expansion ideas, as well as conflict detection scores (i.e., differential evaluations of ideas depending on whether they reflected fixation or expansion). However, one discrepancy emerged regarding the proportion of fixation vs. expansion ideas, which will be discussed below.

As expected, the generative AI demonstrated considerably higher fluency than humans, producing a greater overall number of ideas. Additionally, it explored a greater number of categories than human participants, indicating higher category diversity.

In absolute terms, ChatGPT generated a higher number of both fixation-biassed and expansive creative ideas compared to humans. In other words, while it produces more conventional ideas, it also generates a greater quantity of original ideas. The findings indicate that ChatGPT's outputs exhibit patterns consistent with the fixation bias, similar to those observed in human responses: the majority of its ideas fall into conventional categories (i.e., fixation) and therefore lack originality. When compared to human participants, the individual-level approach revealed that the proportion of fixation ideas generated by ChatGPT was similar to that observed in the 47 human participants. In contrast, the aggregate-level comparison showed that ChatGPT produced a higher proportion of fixation ideas than the weighted average from prior studies, suggesting a stronger fixation bias at the broader, normative level.

Moreover, the ability to simulate a critical evaluation of the ideas being generated seems essential for effectively anticipating phases of convergence or idea filtering when the context requires it. While divergent thinking, characterised by generating a large number of ideas, can foster originality and creativity (e.g., Runco and Jaeger, 2012), it is crucial to identify ideas with genuine added value, particularly those that differentiate from competitors and contribute to innovative solutions. When assessing the model's capacity for differential evaluation of its own ideas, it becomes clear that ChatGPT struggles to distinguish between genuinely original (i.e., expansive) ideas and more conventional (i.e., fixation-based) ones. Unlike humans, who typically perceive original ideas as more creative (e.g., Camarda et al., 2024), the model treats both types of ideas as equally creative.

### 4.3 Interpretation and discussion of the results

The higher fluency score observed in ChatGPT-4o, relative to human participants, reflects its computational capacity to generate a large volume of ideas rapidly and without the cognitive limitations that typically affect human ideation, such as working memory load, mental fatigue, or attentional fluctuations (e.g., De Dreu et al., 2012; Lu et al., 2022; Gong et al., 2023; Gerver et al., 2023). This enhanced fluency highlights the model's ability to traverse semantic networks efficiently and retrieve conceptually relevant outputs from its training data, enabling it to produce a high quantity of responses with minimal delay. Beyond fluency, ChatGPT-4o also demonstrated greater category diversity compared to human participants, producing ideas across a wider range of semantic fields. This category diversity likely stems from its large-scale training on diverse textual data and vector-based semantic representations (OpenAI, n.d.). Such broader exploration, suggesting that ChatGPT-4o can form more distant associations between concepts, may help the model overcome the human fixation bias by generating unconventional, creative ideas (i.e., expansion ideas).

Consistent with this, results showed a higher number of expansion ideas than human participants. These findings are particularly noteworthy, as they highlight the potential value of using generative AI in creative problem-solving contexts. Indeed, a tool like ChatGPT-4o can produce a larger number of creative ideas in a significantly shorter time than humans. However, these creative ideas are embedded within a broader set of mostly conventional responses.

Indeed, ChatGPT-4o generated more fixation-based ideas than expansion ideas, demonstrating a fixation bias comparable to that found in humans (e.g., Agogué et al., 2014; Finke et al., 1992; Smith et al., 1995; Ward et al., 2004; Cassotti et al., 2016; Desdevises and Cassotti, 2024). Supporting this interpretation, the proportion of fixation to expansion ideas did not significantly differ between the AI and human participants, suggesting that while ChatGPT-4o is more prolific, its generative process remains similarly constrained by dominant associations. This pattern may reflect a tendency towards exhaustive generation rather than selective or originality-oriented ideation. The presence of a fixation bias in ChatGPT-4o may stem from the nature of its training data, which predominantly reflects conventional and frequently occurring patterns of human language and reasoning (OpenAI, n.d.). This could lead the model to reproduce dominant associations rather than explore novel or unconventional solutions. However, it remains unclear whether this bias results:

- Directly from the nature of the human-like data on which the model was trained (i.e., namely, large text corpora where frequently repeated associations tend to outweigh rare or unconventional ideas; OpenAI, n.d.)
- or emerge intrinsically from the large-scale pattern learning that underlies generative models, particularly the tendency to prioritise statistically frequent continuations (OpenAI, n.d.) over unlikely but potentially creative associations.

To further investigate the origins of the fixation bias in ChatGPT, a follow-up study could employ controlled prompts that explicitly encourage originality and the rejection of conventional ideas. Comparing responses generated under this more explicit instruction could help determine whether the fixation effect can be modulated by prompt design. A reduction in fixation under creativity-promoting conditions, along with rejection of conventional ideas, would suggest that the bias is not solely inherited from training data but may also arise from the interaction between input framing and the model's generative strategies. However, generating creative ideas is only part of the equation: for a prompt-based strategy to effectively guide ChatGPT-4o towards more original responses, the model must first be able to distinguish between fixation-based and expansion ideas. This distinction is a prerequisite for any filtering mechanism to selectively promote originality. If the model cannot tell the difference, even a prompt explicitly designed to filterfor creative ideas cannot ensure the preferential generation of original responses.

Related to this, my results contradict the assumption made by Guzik et al. (2023) that GPT-4 may be capable of distinguishing between conventional and more novel outputs. Indeed, my findings indicate that the model does not reliably differentiate between fixation-based and expansion ideas, instead generating comparable simulated evaluations of creativity for both types of ideas. These findings reinforce the idea that the fixation bias of GPT-4o is not simply due to its exhaustive strategy and a lack of spontaneous filtering. If that were the case, prompt engineering could plausibly compensate by instructing the model to ignore conventional ideas and prioritise originality. However, given the model's inability to distinguish between original and fixation-based ideas, manipulating the prompt to explicitly promote creativity or to filter out conventional responses would likely remain ineffective. Without a capacity for internal discrimination between idea types, such instructions cannot reliably guide the model towards preferentially generating creative outputs. Thus, to fully harness the capabilities of a generative AI like ChatGPT, two potential avenues for future research seem worth exploring: first, it seems to be crucial to examine whether humans are able to reliably assess and select the most creative ideas among those produced by AI, thereby serving as effective external evaluators. To this end, future studies could benefit from independent human ratings of both the originality and the feasibility of AI-generated ideas. While feasibility was not the primary focus of the present study, it is increasingly recognised as a relevant dimension in creativity assessment, particularly in applied contexts, even though it is not always treated as a core criterion in traditional psychological definitions (e.g., Amabile, 1982). Classic frameworks generally define creativity as the combination of novelty and appropriateness, with the latter sometimes encompassing usefulness, impact, or feasibility in varying degrees. Including feasibility as a distinct rating criterion could thus enrich future evaluations by offering a more nuanced and ecologically valid assessment of AI creativity. Moreover, manipulating transparency regarding the source of ideas (AI vs. human) could help reveal potential biases in creativity evaluation. This line of research would also offer an interesting complementary perspective to current probabilistic approaches, by grounding the evaluation of AI creativity in human-centred, multidimensional appraisal criteria. Second, researchers should investigate whether prompts informed by key theoretical principles; such as C-K theory, the importance of distant semantic associations, or the model's own probabilistic mechanisms; could strengthen its capacity to more accurately evaluate and filter the ideas it generates. Such strategies may not eliminate the fixation bias, but they could help shape outputs in ways that better align with creative task demands.

Beyond the technical and methodological considerations, it is also important to emphasise that ChatGPT-4o is not an autonomous creative agent. It lacks consciousness, intentionality, and genuine metacognitive capacities (e.g., Bai et al., 2025; Zhou and Lee, 2024); its outputs reflect only probabilistic inferences over patterns learned from data. Accordingly, in real-world creative contexts, the most valid perspective is to regard such models as collaborative tools rather than independent creators. This human–AI collaboration implies a synergy between the respective strengths of both: the model's capacity to rapidly generate diverse and semantically rich ideas, and the human ability to critically evaluate, select, and refine the outputs within meaningful and context-sensitive frameworks. However, this collaboration also poses challenges, including the risk of over-reliance on AI suggestions and the absence of contextual or ethical judgment on the part of the model. Therefore, future work should continue to explore not only how to improve generative performance but also how to optimise human–AI interaction (e.g., Fang et al., 2025; McCormack et al., 2020), in particular to support responsible and genuinely creative outcomes (Pflanzer et al., 2023; Vinchon et al., 2023; Vössing et al., 2022).

### 4.4 Limitations and future directions

Several methodological limitations should be acknowledged and may guide future experimental improvements.

First, although the main analyses were based on 50 bootstrapped observations generated by ChatGPT-4o, all outputs stem from a single underlying model instance (ChatGPT-4o). As explained in the Methodology, multiple generations from the same model capture its inherent stochastic variability and represent plausible outputs under fixed prompt conditions. While this approach aligns with recent practises in computational research (e.g., Chen et al., 2021; Binz and Schulz, 2023), it nonetheless limits the generalisability of findings. Recent research indicates that these repeated outputs can in some cases be correlated, potentially limiting the effective diversity of generations and necessitating caution when interpreting variability and statistical inference (Gallo et al., 2025). Future studies could improve this by including outputs from different architectures, model versions, or independently trained instances, thereby offering a broader representation of AI-generated variability. Additionally, although 50 bootstrapped observations provide a reasonable basis for inference, increasing this number, alongside the number of human participants, could further strengthen statistical power. Moreover, future research should consider applying statistical approaches that account for correlations between repeated outputs of ChatGPT, such as mixed-effects models or dependency-adjusted resampling techniques, to yield more reliable estimates of variability (Gallo et al., 2025).

Second, the use of aggregated human data from prior studies was intended as an exploratory, normative benchmark rather than a basis for inferential comparison. The analysis relied on weighted mean scores from eight published experiments using the same task and participant profile. However, the unavailability of individual-level data limits the capacity to capture intra-study variability and precludes a full meta-analytical treatment. While these weighted averages help contextualise the AI's performance within a normative human range, a more rigorous meta-analysis using original datasets would provide a clearer and statistically grounded picture of typical human performance on the task.

Third, this study focused on a single creative task (i.e., the egg task) which, while well-suited for measuring divergent thinking and idea generation, may limit the generalisability of the findings. Creativity is a multifaceted construct that manifests differently across domains and tasks. Further research should replicate and extend this approach using a broader range of tasks that tap into other dimensions of creativity, such as analogical reasoning, storytelling, or design innovation for instance.

## 5 Conclusion

The findings of this study underscore the significant potential of generative models like ChatGPT-4o in accelerating both the quantity and creativity of ideation and content generation. However, it is clear that such tools should not be regarded as autonomous creative agents. While they demonstrate impressive fluency and the ability to produce a large number of creative ideas, their inability to critically assess the originality of their own outputs reveals a key limitation. As highlighted by Kumar et al. (2024), over-relying on AI in creative processes can pose risks. Therefore, ChatGPT-4o and similar models should be viewed as cognitive assistants, powerful yet requiring human oversight. Rather than replacing human creativity, these tools are best utilised to support and enhance the ideation process, with users critically evaluating and refining the generated ideas.

## Data Availability

The datasets presented in this study can be found in online repositories. The names of the repository/repositories and accession number(s) can be found below: https://osf.io/cqpmr/?view_only=ef11630f521a4cdd95dd9200e1c7ea40.
